# Medical malpractice regulation. Civil, administrative, and criminal liability

**Published:** 2018

**Authors:** Ionuţ Ciprian Săraru

**Affiliations:** * Săraru Ionuţ Ciprian Law Office, Bucharest, Romania

**Keywords:** malpractice, liability, damages, reuse, consent, information, confidentiality, medical devices

## Abstract

The evolution of the modern Romanian society reveals an increasing trend as to the liability for medical malpractice, which in correlation with recent amendments of relevant legislation should make healthcare professionals increase their awareness in this matter. In the analysis below, malpractice was defined from a theoretical and practical perspective, and approached from its potential consequences. As liability for malpractice could be variable subject to the nature and importance of the damages incurred, the analysis herein describes the civil, administrative, and criminal liability in general, as well as the main causes for the occurrence of malpractice (e.g. professional errors, lack of information and consent of the patients, reuse of single-use medical devices, etc.). Furthermore, a few recommendations have been further provided in order to eliminate or diminish malpractice from the healthcare professionals and medical institutions day-to-day activity.

All medical activity requires not only a very solid education and training, but also a vigilant and open mind towards its legal regulation and consequences for breaching them. This subject should be given the same importance as to the preparing for the medical activity, considering the complexity of the legislation with its sometimes-contradictory interpretation. 

The importance of embracing discussion of malpractice matters lie in the current status of medical activities, the evolution of the Romanian society, the formalization of the patient-doctor relationship, and the increasing level of awareness as regards the patients’ fundamental rights. 

The specific regulation that should be considered is Title XVI of the Law no. 95/ 2006 regarding the health reform (the “Health Reform Law”) together with its application norms, while the Romanian Civil Code and the Romanian Criminal Code provide for the consequences of malpractice.

Malpractice could be defined as the professional error that produces a prejudice to the patient, which may trigger civil, administrative, and criminal liability [**1**]. 

As regards the *error*, it is important to know that the liability is generated by two types of errors: (i) errors against medical science, that include professional errors, tort, insufficient medical knowledge, individual acts within the prevention, diagnosis and treatment process (e.g. performing experiments, reuse of single-use medical devices etc.) and (ii) errors against medical conscience that include, among others, the breach of confidentiality and the lack of patients’ informed consent for the medical assistance [**2**].

The subjects of medical malpractice include a large category of healthcare professionals (e.g. doctors, pharmacists, nurses), as well as private and public medical institutions and manufacturers of medicines and medical devices. 

As regards the *consequences* of malpractice, three types of *liability* have been identified: (i) the civil liability leading to the indemnification of the patients for their damages, (ii) the administrative liability leading to disciplinary/ administrative penalties for the healthcare professional and (iii) the criminal liability which triggers criminal sanctions to the healthcare professionals in case of actions qualified as offences by the criminal law (e.g. bodily harm, manslaughter, etc.).

For the existence of liability, the following conditions should be cumulatively met and proven: (i) the existence of an illicit act, (ii) the existence of damage, (iii) the existence of guilt and (iv) the existence of the cause-effect report between the illicit act and the damage [**3**].

It is important to note that in a malpractice case there may be also a joint (common) liability of the healthcare professional with the medical institution where such case occurred [**4**]. At the same time, it is important to note the lack of liability for malpractice when the healthcare professional acts with good faith in emergency cases and when the damage occurs due to the working conditions, insufficient endowment of the medical institution, nosocomial infections etc. [**5**].

Besides the professional error in treatments or particular interventions, in practice there have been identified specific situations that can generate liability. The most important that should be considered are the following:

1. Lack of patient’s informed consent on: (i) the diagnosis, treatment, the result of the treatment and the consequences for not getting the treatment, (ii) the risks of an intervention or a treatment as well as the alternatives to the treatment with their underlying risks [**6**]. 

Considering the consequences of a treatment/ intervention or their alternatives, a special attention should be given to the information part of the medical act, at the same time ensuring that the patient understood such consequences in order to decide on accepting a treatment or intervention. Such information should be given in writing followed by the patient’s signature. In practice, healthcare professionals may encounter cases in which patients have a lack of discernment or a low capacity of understanding. These cases should be carefully carried out by specifying the particular situation of the patient further to its complete information.

2. Reuse of single-use medical devices. In these cases, not only direct interdictions to reuse the single-use medical devices are breached (regulated by the Ministry of Health Order no. 961/2016 [**7**] and Government Decision no. 54/2009 [**8**]), but also deontological norms and fundamental rights of patients to physical integrity and security. 

It is important to know that in these particular cases the liability triggered by a malpractice could be higher compared to the perpetration of a classical professional error. Such increased liability derives from the intrinsic nature of this action, considering that the healthcare professional is aware of the consequences or the reuse of single-use devices, which are disregarded. It should be noted that the liability for these particular cases could trigger more severe consequences for the healthcare professional and the medical institution. 

3. Breach of doctor – patient confidentiality in the activity to promote the performance of specific state of the art procedures or new techniques in surgical interventions [**9**]. In this respect, there may be cases in which medical institutions promote specific cases on social media for marketing purposes, without the prior consent of the patient for the use of their image. 

4. Other situations triggering liability of healthcare institutions and professionals resides in the lack of clear working and communication procedures, which, in correlation with understaffed medical teams, may contribute to the perpetration of professional errors.

Considering the particular activity of medical institutions, on one hand, malpractice could be avoided through continuous training and education of healthcare professionals, which are direct obligations in this area. On the other hand, strengthening the awareness on the importance of patient information and the forms used by the medical institution together with the improvement of operational flows could diminish the occurrence of malpractice. 

Other methods to avoid malpractice cases is the enforcing of internal policies for the elimination of the reuse of the single-use medical devices, not only by increasing the healthcare professionals awareness on its consequences, but also by enforcing internal procedures regulating the single-use of such medical devices. This measure goes hand in hand with the revising of the employment documentation used in relation to the healthcare professionals (employment agreements, job descriptions, services agreements, internal regulations, etc.). 

As regards the consequences of the civil liability that triggers indemnification of the patients for damages caused by malpractice, reassessment of the civil liability insurance policies should be considered, not only from the insured amount perspective, but also from their general terms and conditions. 

To conclude, malpractice regulation, and familiarity with it should be afforded the greatest importance considering the consequences that may be triggered against healthcare professionals, medical institutions and the other subjects involved in the medical act. In this respect, healthcare professionals should be aware of malpractice regulation starting at an early stage of their career, preferably even in medical school, considering the specificity of such regulation and its consequences for themselves and for the patients.
